# Rational Design of Solid Polymer Electrolyte Based on Ionic Liquid Monomer for Supercapacitor Applications via Molecular Dynamics Study

**DOI:** 10.3390/polym14235106

**Published:** 2022-11-24

**Authors:** Baris Demir, Kit-Ying Chan, Sébastien Livi

**Affiliations:** 1 Centre for Theoretical and Computational Molecular Science, The Australian Institute for Bioengineering and Nanotechnology, The University of Queensland, Brisbane, QLD 4072, Australia; 2 Department of Aeronautical and Aviation Engineering, The Hong Kong Polytechnic University, Kowloon, Hong Kong SAR, China; 3 Ingénierie des Matériaux Polyméres, Université de Lyon, CNRS, UMR 5223, INSA Lyon, F-69621 Villeurbanne, France

**Keywords:** ionic liquid, solid polymer electrolytes, molecular dynamics simulations, thermo-mechanical properties

## Abstract

The safety concern arising from flammable liquid electrolytes used in batteries and supercapacitors drives technological advances in solid polymer electrolytes (SPEs) in which flammable organic solvents are absent. However, there is always a trade-off between the ionic conductivity and mechanical properties of SPEs due to the lack of interaction between the ionic liquid and polymer resin. The inadequate understanding of SPEs also limits their future exploitation and applications. Herein, we provide a complete approach to develop a new SPE, consisting of a cation (monomer), anion and hardener from ions–monomers using molecular dynamics (MD) simulations. The results show that the strong solid–liquid interactions between the SPE and graphene electrode lead to a very small gap of ∼5.5 Å between the components of SPE and electrode, resulting in a structured solid-to-liquid interface, which can potentially improve energy storage performance. The results also indicated the critical role of the mobility of free-standing anions in the SPE network to achieve high ionic conductivity for applications requiring fast charge/discharge. In addition, the formations of hardener-depleted regions and cation–anion-poor/rich regions near the uncharged/charged electrode surfaces were observed at the molecular level, providing insights for rationally designing the SPEs to overcome the boundaries for further breakthroughs in energy storage technology.

## 1. Introduction

To reduce the carbon footprint of fossil fuels, electric transportation, especially electric vehicles (EVs), has pursued the rapid development of electrochemical energy storage devices, including batteries, supercapacitors and conventional dielectric capacitors. Among them, supercapacitors have exhibited superior power density, fast charge and discharge rate and long lifespan [1,2], showing high potential to be the next-generation energy storage technology. Liquid electrolytes (LEs) are preferably used in traditional supercapacitors because of their high ionic conductivity and wettability to the electrode surface [3]. However, the risk of triggering a fire due to the presence of flammable organic solvents in LEs raises the safety concerns. Solid polymer electrolytes (SPEs), in which flammable organic solvents are absent, are promising multi-functional energy storage applications thanks to their superior mechanical robustness and chemical stability. The micro-porous separators, which are commonly polyethylene (PE)- and polypropylene (PP)-based, in Li-ion batteries are used for separating the anode and cathode to prevent a short circuit. In this case, electrolytes still need to be used for high-energy storage performance. By contrast, SPEs can be fabricated into membranes to provide ion conduction while separating the electrodes [4,5]. The absence of a conventional separator will lead to better energy storage [6]. However, the low ionic conductivity (∼10${}^{-5}$ S cm^−1^) of SPEs hinders their wide application [7].

Ionic liquids (ILs) are key materials for improving the energy-storage performance and flexibility of various electrochemical devices, such as batteries and sensors [8,9,10,11]. Among SPEs, epoxy polymers based on novel ILs are promising materials, as they possess similar thermo-mechanical properties to the oil-derived counterparts (e.g., DETDA) and additional properties, including ionic conductivity [12,13] and hydrophobicity [14,15]. Aspects such as chemical/electrochemical stability and thermo-mechanical strength are important when choosing SPE materials. It is noteworthy to note that there is always a trade-off between mechanical robustness and energy storage performance in SPEs, requiring more fundamental studies to optimize their overall performance.

Kwon et al. [16] indicated that a concentration of 70 vol% IL in a cross-linked epoxy matrix system provided the highest conductivity but at the expense of Young’s modulus, highlighting the plasticizing effect of IL and the conductivity imparted by the IL. Matsumoto and Endo [17] reported the importance of the extent of IL confinement in the matrix and its crucial role on the ionic conductivity of the polymer matrix, highlighting the various aspects in determining the ultimate performance of SPE. In another work, Yao et al. [18] revealed that the molecular structures of an SPE comprised of glycidyl methacrylate (GMA) and oligo(ethylene oxide) methyl ether methacrylate (OE) affected the diffusivity of Li+ ions. The epoxy group included at the distal end of the GMA monomers in the polymer chains imparted smaller conductivity when compared to the polymer network, where the hydrolysed form of the GMA monomer (where the epoxide bond is broken) is used. More recently, Song et al. [19] reported that the formation of diffusion channels and morphologies of an SPE, which was made of a cross-linked epoxy matrix integrated with ionic liquid and/or Li salt, varied in length scales as a function of the salt concentration in the polymer matrix, which helped regulate the diffusion of ions to alter the ionic conductivity. In the aforementioned work, both the anion and cation are free to move within the samples and they do not form a chemically linked polymer network. As such, the lack of interactions between the two separated phases (i.e., epoxy and ILs) becomes the bottleneck in achieving both high mechanical and electrical performance for SPEs. Therefore, it is highly desirable to develop a novel SPE with high ionic conductivity and mechanical robustness after polymerisation.

Thanks to the increase in computational power, computer tools such as density functional theory (DFT) calculations and molecular dynamics (MD) simulations are useful means to fully capture the atomistic and molecular-level behaviour of materials, including pure polymers [20,21,22] and polymer mixtures [23], polymer composites [24], solid-state electrolytes [25,26] and supercapacitors [9]. Despite numerous experimental works on the investigation of SPEs, the number of computational studies focusing on the elucidation of the molecular-level behaviour of the IL-polymer matrix as SPE used in supercapacitor models is limited. Recently, Demir et al. [27] devised a computational protocol to study the effect of IL concentration on various aspects including the phase separation between the IL and epoxy matrix, plasticisation, thermo-mechanical properties (i.e., glass transition temperature and Young’s modulus) and ionic conductivity using MD simulations. In addition, Demir and Searles investigated the behaviour of two ILs confined in between graphene electrodes as a response to the potential difference created across the supercapacitor model [9]. They showed that various regions, such as cation–anion-poor/rich regions formed due to the potential difference. Additionally, the extent of the exclusion/inclusion of ions with opposite charges increased with increasing the applied potential difference.

Ionic liquid monomers (ILMs) [28] have been shown to be promising smart and multifunctional building blocks for polymers used in structural applications due to improved macroscopic properties, including thermo-mechanical performance [29,30], and health applications due to antibacterial [31,32] and self-healing properties [33,34,35]. The polymerisable nature of ILMs allows the formation of polymer networks between ILMs and the epoxy network through copolymerisation, exhibiting excellent thermal and mechanical stability [30]. However, there is still a lack of molecular-level investigations on macroscopic properties of ILM-based SPEs in a supercapacitor’s system, where a constant potential is applied, using MD simulation. In this work, ILMs (or the cation or monomer) and hardener (or cross-linking agent) (isophorone diamine) chemically take part of the epoxy network in which the cation compensates the charge neutrality and acts as co-monomer for the epoxy–amine network to form a novel SPE.

Molecular-level investigations of a supercapacitor, which is made by graphene electrodes and the novel SPE, have been carried out to study the distribution of components in precursor mixtures and after polymerisation as well as under different potential differences and temperatures. This work offers a complete approach to develop robust SPEs using ILMs, opening up new opportunities toward meeting the need for high-performance solid electrolytes for supercapacitor applications. It also demonstrates that MD can provide insights into the rational design of new-generation electrolytes with both high mechanical performance and high ionic conductivity.

## 2. Methods and Simulation Procedure

### 2.1. General Simulation Details

All-atom DREIDING force field (FF) (with the Lennard-Jones (LJ) form) was used to calculate the potential energy of the samples studied here [36]. DREIDING FF is a suitable FF, as it has been tested on pure ILs and poly(ionic liquids) (PILs) [37,38,39,40], and neat epoxy systems [20,21] as well as their composites [41,42,43,44]. Periodic boundary conditions in all three dimensions were implemented. The Nosé–Hoover thermostat [45,46] (in both the NVT-MD and NPT-MD simulations) and Nosé–Hoover barostat [45,47] (in the NPT-MD simulations) were used to control the temperature and pressure of the samples, respectively. A cut-off distance of 12 Å was used for long-range energy calculations. A particle–particle–particle–mesh (PPPM) solver was implemented for calculating the contribution of long-range interactions [48]. A time-step of 1 fs was used throughout this work. MD simulations were performed using the open-source molecular simulation tool, LAMMPS (lammps.sandia.gov) [49]. System trajectories were visualised using Visual Molecular Dynamics (VMD) [50].

### 2.2. Sample Preparation Procedure

**Figure 1:**
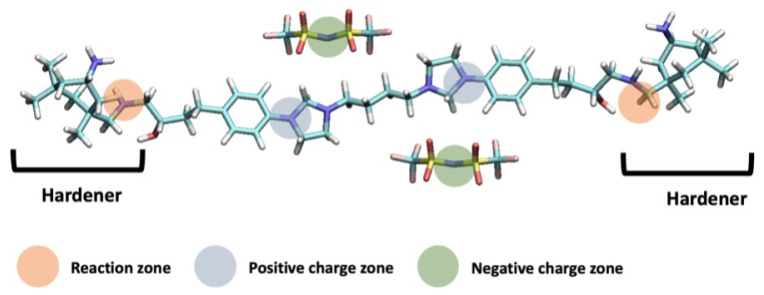
A representation of the reaction between one cation and two hardeners as shown in full orange circles. The negative charge zone on the anions and positive charge zone on the cations are shown as full green and grey circles, respectively.

The liquid precursor mixture is composed of an IL based on a bis-imidazolium cation bearing two epoxy rings (carrying a net charge of +2), bis(trifluoromethanesulfon yl)imide (anion) (carrying a net charge of −1) and a hardener, isophorone diamine (IPD) (carrying a net charge of 0), as shown in Figure 1. The initial structure of the three components was generated using the AVOGADRO software package [51]. An initial simulation cell with a dimension of 41.76 Å, 42.54 Å and 180 Å in the x-, y- and z-directions, respectively, was generated, which included a graphene layer located at both ends in the z-direction. The x- and y-directions of the electrode were selected so that they are at least two times larger than the size of any component of the electrode. The bulk density of the solid polymer electrolyte was considered to adjust the size of the z-direction. In total, 192 cations, 384 anions and 96 hardeners were randomly placed between the graphene layers in the simulation cell using PACKMOL [52]. The electrode-to-electrode distance in the z-direction was chosen accordingly so that the density of the electrolyte was ∼1.13 g·cm^−3^ [30]. All the components, except the graphene electrodes, were kept flexible in space during the simulations. The partial atomic charges of the atoms of each component are reported in the literature [30].

The liquid precursor mixture that was placed in between the graphene layers was equilibrated at 800 K for 20 ns via the use of NVT-MD simulations. At the end of each 4 ns, a 200 ps simulation was performed to generate a trajectory for further analysis. The reason for using such an elevated temperature was to ensure a proper mixing of the species (i.e., anion/cation and hardener) so that the polymerisation can happen uniformly everywhere in the simulation cell.

### 2.3. In Situ Crosslinking Procedure

**Figure 2:**
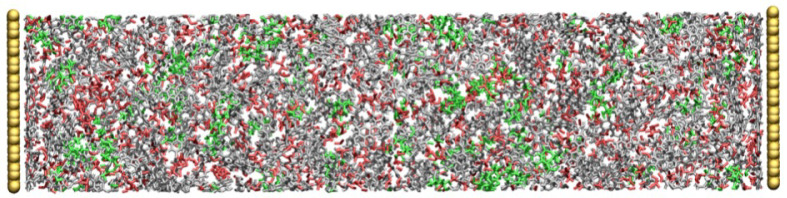
The supercapacitor model after the completion of the polymerisation procedure. Colour scheme: grey, red, green and yellow are for cation, anion, hardener and graphene electrode, respectively.

A crosslinking procedure previously reported in the literature [21,30] was followed. The bonds between the reactive atomic sites, i.e., the carbon atom located on the distal ends of the cation and the nitrogen atoms of the hardener, were captured on-the-fly. Similar to the published work, a polymerisation temperature of 500 K was used [21,30]. Figure 2 shows a supercapacitor system with the polymerised IL. The polymerisation procedure was terminated when the target degree of crosslinking (DOC) was achieved (i.e., 80%). Upon the completion of the polymerisation procedure, we updated the topology of the polymerised system by deleting the excess hydrogen atoms that were chemically connected to the reacted carbon atoms of the cation and reacted nitrogen atoms of the hardener (Figure 1). The system was then equilibrated over a period of 1 ns in the NVT-MD ensemble at three temperatures: 300 K, 400 K and 500 K.

### 2.4. Constant Potential Simulations

Once the equilibration simulations were completed, a vacuum region of 20 Å was added on both sides of the simulation cell beyond the fixed electrodes in the z-direction, and the system was kept periodic in the x- and y-directions [9]. A PPPM solver [48] with slab geometry imposed using the Yeh–Berkowitz condition was used to truly capture the 2D periodic boundary conditions [53]. A constant potential difference was applied across the supercapacitor model using the constant potential method (CPM) [54,55,56] implemented in LAMMPS [49]. The CPM allows the partial atomic charges of the electrode atoms to fluctuate as a response to change in the concentration and composition of the electrolyte in the electric double layer (EDL), which is the region of electrolytes that is immediately affected by the surface polarisation. The thickness of EDL can be up to several nanometres [57]. What distinguishes the CPM method from the fixed-charge method (FCM) is that the partial atomic charges of the electrode atoms are fixed in the latter. In the CPM method, the charges of the electrode atoms are assumed to be located with a Gaussian distribution centred on the atoms. A Gaussian parameter of 19.79 nm^−1^ was used [54]. The partial atomic charges of the atoms of the polymerised electrolytes remained unchanged during the constant potential simulations. A potential difference (Ψ) between 0 and 4 V was applied across the simulation cell, and the behaviour of the polymerised IL was observed as a response to change in Ψ at three temperatures, 300 K, 400 K and 500 K.

## 3. Results and Discussion

All the results reported in this section are averaged over three independently generated samples. The DOC of each sample was fixed to 80%. The results obtained in the liquid electrolyte and polymerised electrolyte (i.e., no potential difference applied) are first presented and discussed. Later, a constant potential difference was applied across the simulation cells at three temperatures. The results obtained in the presence of potential difference are provided starting from the section Charge Density Distribution on the Electrodes.

### 3.1. Mass and Charge Density Distribution in the Liquid Precursor Mixture

**Figure 3:**
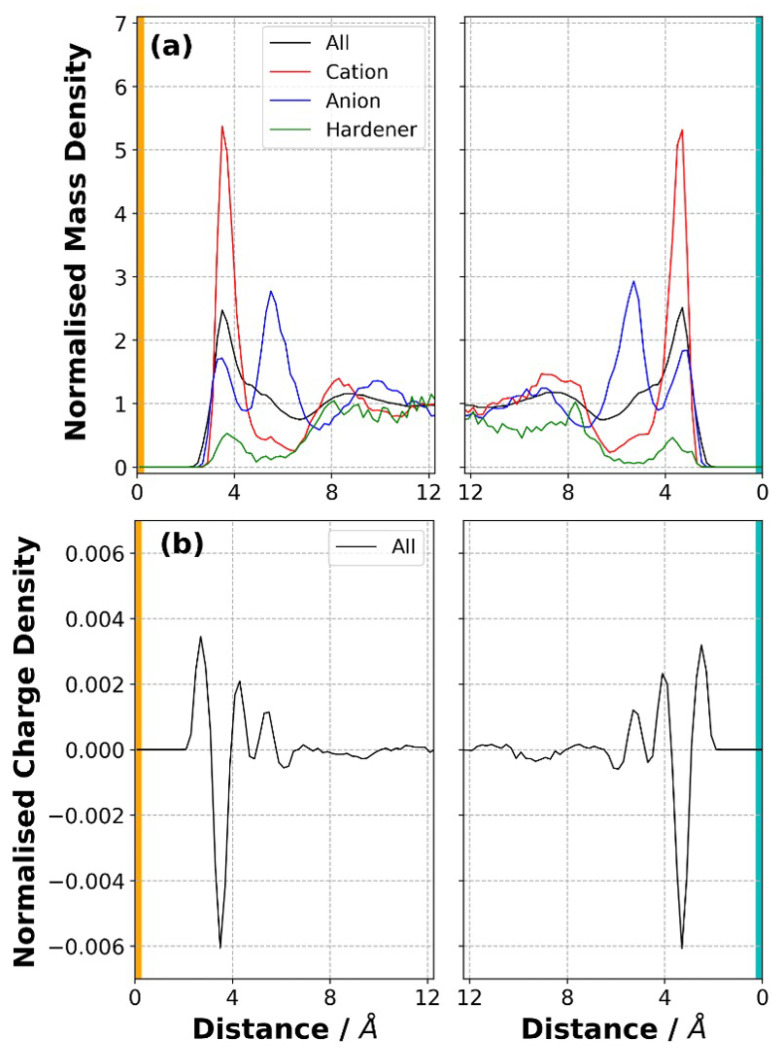
(**a**) Normalised mass density distribution for each component and (**b**) normalised overall charge density distribution near the negative (orange bar) and positive (cyan bar) electrodes obtained at the completion of the equilibration process of the liquid precursor mixture at 800 K. No potential difference was applied across the simulation cell.

Three components of the electrolyte, anion, cation (or monomer) and hardener, were mixed using an NVT-MD simulation at 800 K for 20 ns. N atom of anion, C atom of one of the imidazolium rings of cation and one of the N atoms of hardener (Figure 1) were used as reference points in the calculations of normalised mass density distribution, as shown in Figure 3a. The results indicate that the components were mixed well, and almost symmetrical distributions were obtained for each component. Since the number of hardeners is the smallest one among the three components, it is expected that a noisier normalised mass density distribution curve for the hardener (green curves Figure 3a) will be obtained. It is found that a peak located at ∼3.5 Å away from each electrode appeared for the entire liquid and for each component. This can be attributed to the interactions of components with the solid electrode surfaces. A second peak formed for the anion at ∼5.5 Å. Since the cation carries a net charge twice of the anion, two anions are found near each cation. This can be one of the reasons for the formation of a second peak for the anion away from the electrode surface. Interestingly, a depletion in the distribution of the hardener in the vicinity of electrode surfaces is observed. In other words, the hardener molecules preferred to be found away from the electrode surfaces when no potential difference was applied across the cell. This can be due to the strong Coulombic interactions between the anion and cation, which occupied the region next to each electrode and no available space left for the hardener near the electrode. In addition, the overall charge density distribution in the liquid sample was also calculated (Figure 3b). The results indicate that the nearest peak to each electrode is a positive peak, followed by a larger negative peak. The first positive peak can be attributed to the presence of a cation next to the electrodes. On the other hand, the first negative peak can be due to the combination of partial charges belonging to atoms of the components found in the vicinity of the electrodes.

### 3.2. Mass and Charge Density Distribution in the Polymer

Once the polymerisation process started, the reactive carbon atoms of cation and reactive nitrogen atoms of the hardener formed covalent bonds. Since a smaller number of hardeners and a larger number of cations are observed in the vicinity of electrodes, this will result in varying densities of reacted atoms in the same region. To quantify this, the distribution of the reacted carbon atoms of cations in the normal direction to each electrode surface is calculated. Upon the completion of the polymerisation process, a 1 ns NVT-MD simulation was performed to equilibrate the polymerised samples at three different temperatures, and the distribution of the reactive carbon atoms was calculated using the last 0.2 ns of the simulation, reported in Figure S1a. The distributions calculated at temperatures varying between 300 and 500 K highlight that the polymer solidified during the polymerisation and the reactive carbon atoms of the cations that are making part of the polymer network have a limited mobility. On the other hand, the anions, which do not chemically join the polymer network are free to move in the solid system. The mobility of anions as a response to the applied potential difference will be investigated in the next section. In addition, the solidification of the samples appear not to affect the charge density distribution in the sample (Figure S1b), as the mobility of some components was limited.

### 3.3. Charge Density Evolution and Distribution on the Electrodes

**Figure 4:**
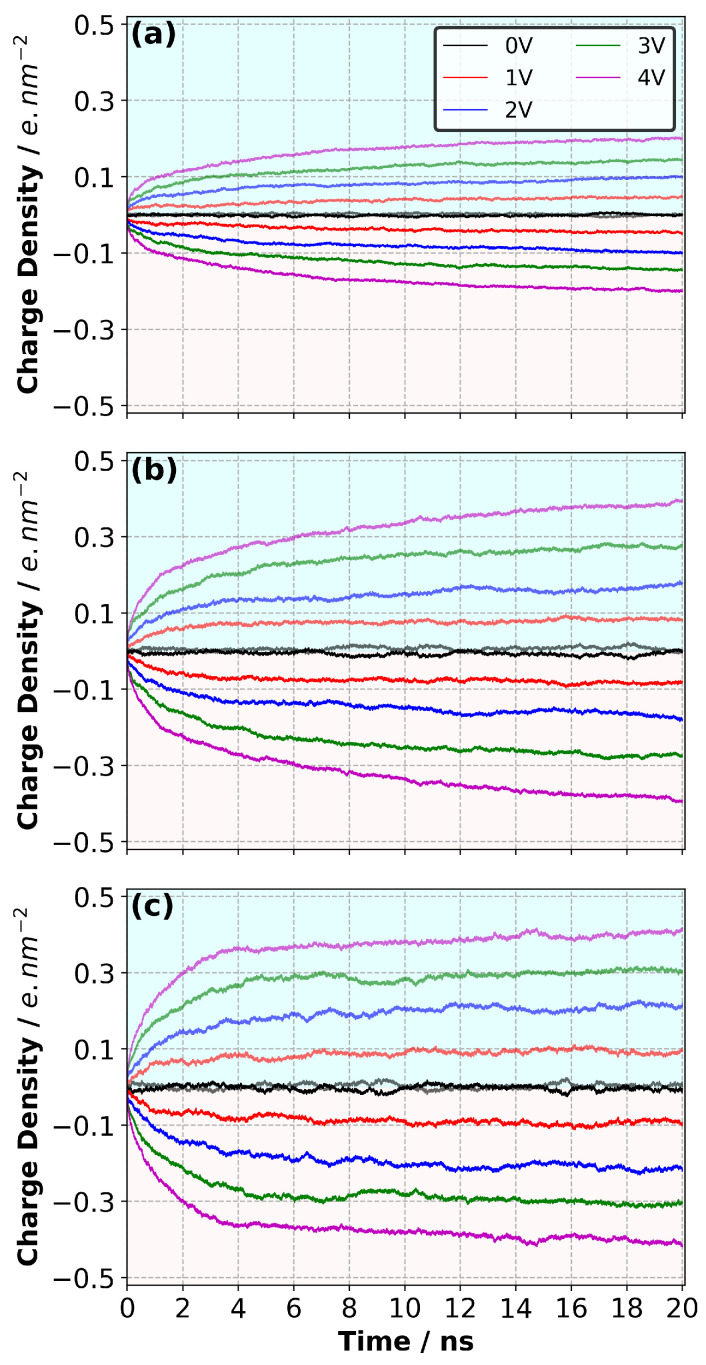
Evolution of charge density distribution on the electrodes as a function of potential difference at (**a**) 300 K, (**b**) 400 K and (**c**) 500 K.

Upon the completion of the equilibration of the polymerised samples, a constant potential difference (ΔΨ) with a range of 0 and 4 V was applied across the supercapacitor model at three temperatures. The results indicate that a duration of 20 ns simulation with applied potential was sufficient to obtain the equilibrium charge density on each electrode for the range of ΔΨ studied here, as shown in Figure 4. As expected, the ultimate absolute value of the charge density on each electrode increased with an increase in ΔΨ. This is caused by the reorientation/accumulation of ions in the EDL [9]. The ultimate absolute value of the charge density at a fixed ΔΨ increased with the increase in temperature. The difference was larger when the temperature was increased from 300 K to 400 K, whereas it was smaller when the results that were obtained at 400 K and 500 K, were compared. In addition, with the increase in the temperature, the system came to saturation faster because the slope of the CDD curves flattened out faster when the temperature increased. In particular, at 500 K, 5 ns of simulations with potential difference was sufficient to charge the electrodes almost fully, whereas a time of ∼15 ns was needed to achieve this at 300 K. The results indicate that the temperature facilitated the mobility of ions and caused the electrode to fully charge within a shorter simulation time (Figure 4).

**Figure 5:**
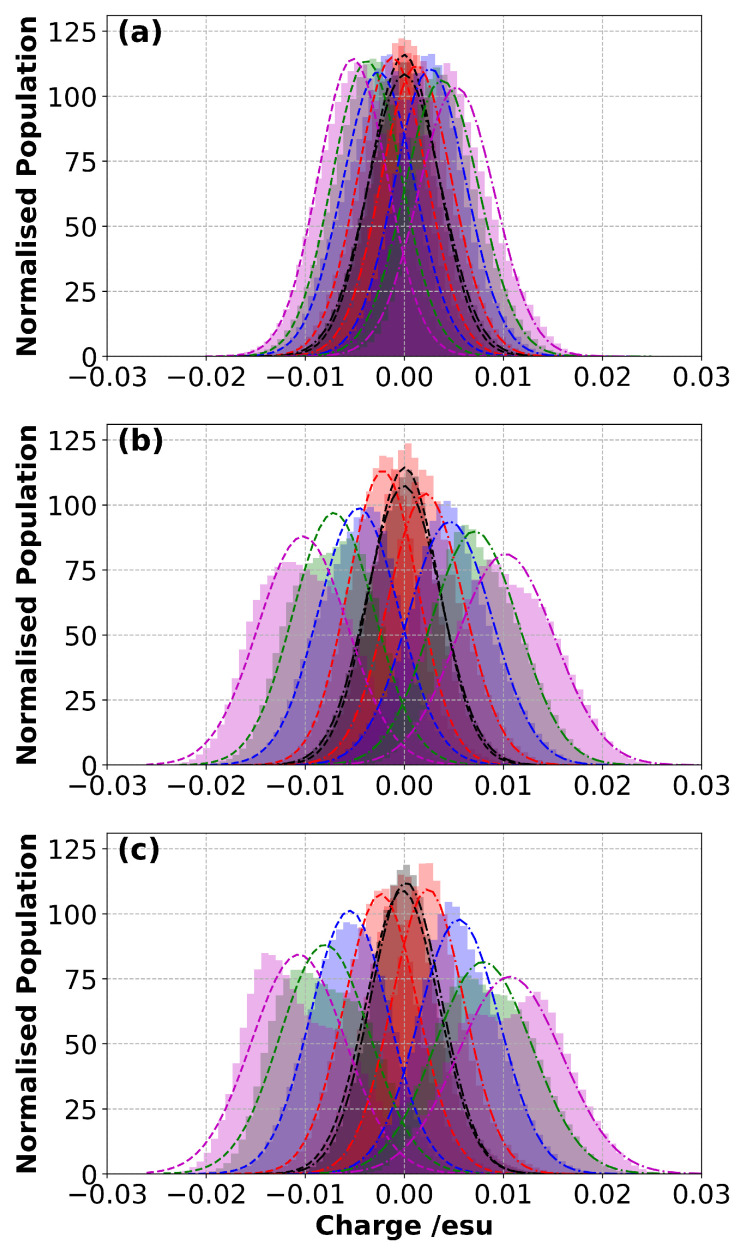
Probability distribution of partial atomic charges of the electrode atoms in response to ΔΨ at (**a**) 300 K, (**b**) 400 K and (**c**) 500 K. Dashed lines represent the Gaussian fit curves. Colour scheme: black, red, blue, green and magenta for 0 V, 1 V, 2 V, 3 V and 4 V respectively.

To capture how the temperature affects the charge distribution on the electrode, the histogram distribution of the partial atomic charges of the electrode atoms, which was developed in response to ΔΨ at different temperatures (Figure 5), was calculated. It was found that at the lowest temperature (300 K), the distribution of the partial atomic charges was narrower, and the peak amplitudes of each curve obtained at different ΔΨ were comparable (Figure 5a). When the temperature was increased to 400 K, it was observed that the distribution of the partial atomic charges widened, but the peak amplitudes decreased together with the increase in ΔΨ (Figure 5b). A further increase in the temperature (from 400 K to 500 K) seemed not to substantially affect the partial charge distribution (Figure 5c) when compared to 400 K. In other words, the change in the histograms was more pronounced between 300 K and 400 K than that between 400 K and 500 K. From these results, it can be concluded that performing simulations at least at 400 K would be beneficial in terms of capturing the dynamics of the SPE for comparison with experimental observations.

### 3.4. Reorganisation of Anions in the EDL

**Figure 6:**
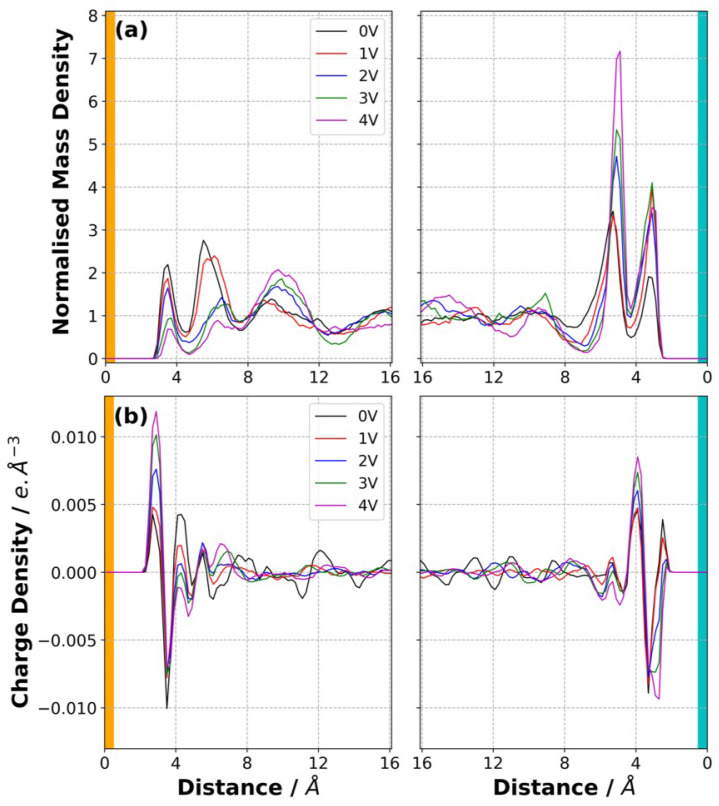
(**a**) Normalised mass density distribution of the anion in the SPE at 500 K at various ΔΨ. (**b**) Charge density distribution of the SPE at 500 K at various ΔΨ.

The normalised MDD of the anion calculated at 500 K and at various ΔΨ is shown in Figure 6a. Our results indicate that the anions were pushed away from the negative electrode in response to increase in ΔΨ. This can be attributed to the unfavoured Coulombic interactions between the negatively charged ions and negatively charged carbon atoms of the electrode (the orange region in Figure 6). Similarly, the anions accumulated near the positive electrode (the cyan region in Figure 6) with ΔΨ. These trends held for all three temperatures studied here (Figure S2).

**Figure 7:**
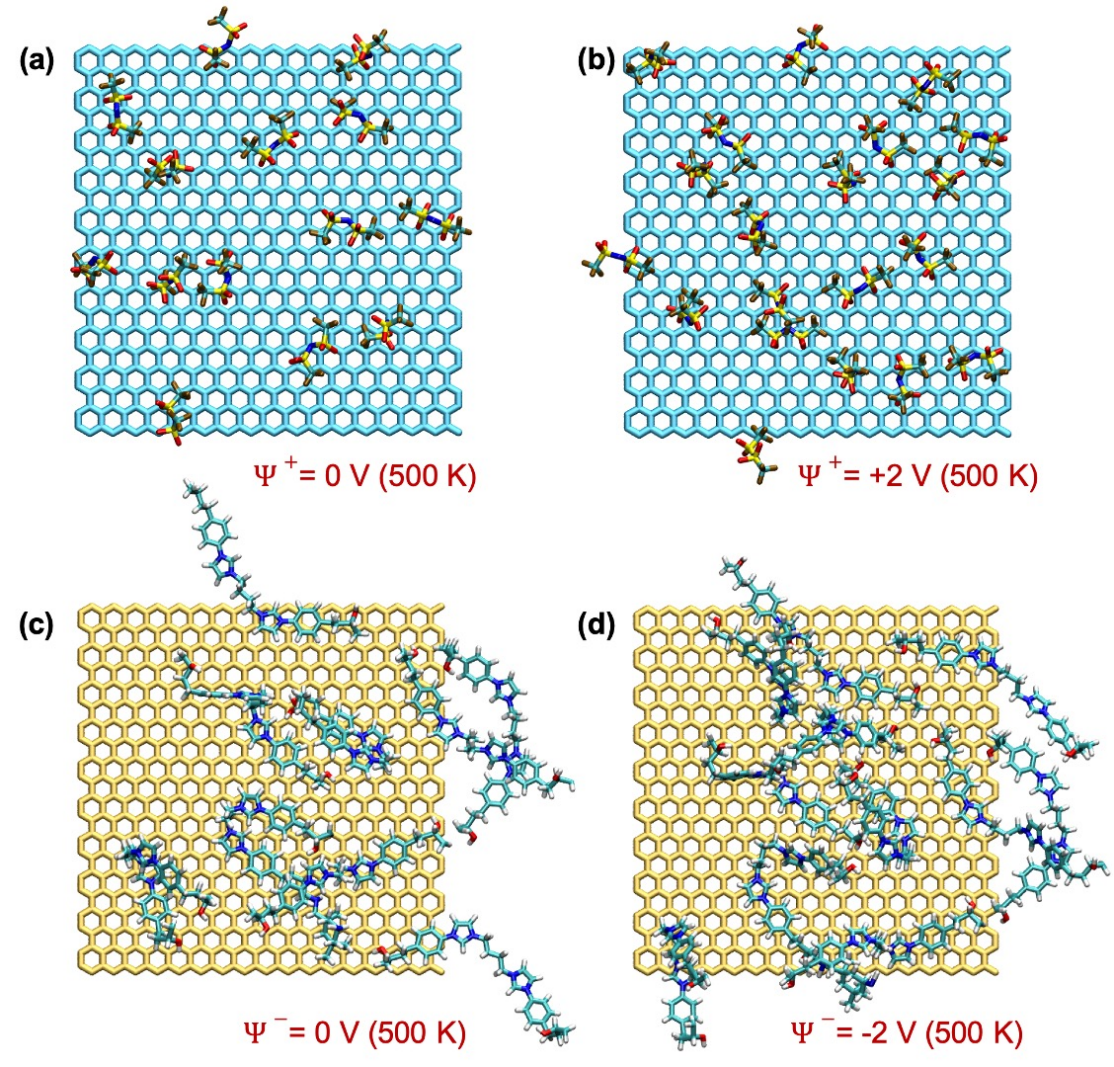
Snapshots taken at 500 K at various ΔΨ showing (**a**,**b**) the anions on the positive electrode and (**c**,**d**) the polymer network on the negative electrode, found within a distance of 6 Å.

It is interesting to note that the increase in the amount of anions in the very first layer from the positive electrode surface stopped substantially at ΔΨ larger than 2 V. This can be due to the fact that the 3D polymer network is not mobile and occupies most of the space next to the positive electrode. However, an increase in the amount of anion in the second anion-rich layer from the positive electrode was observed until the highest ΔΨ applied. Figure 7a,b shows a snapshot of anions found within a 6 Å distance from the positive electrode at 500 K at 0 V and 4 V and indicates the crowding of anions in response to ΔΨ.

The charge density of the SPE, shown in Figure 6b showed a similar trend. The charge density of the first peak near the positive electrode remained almost unaltered in response to ΔΨ, whereas the amplitude of the second peaks kept increasing with ΔΨ. This result suggests that the SPE used for the supercapacitor applications needs to be designed so that the anions will be able to penetrate into the very first few layers formed next to the positive electrode.

In a similar manner, the charge density in the first layer near the negative electrode (Figure 6b) increased with ΔΨ. The normalised MDD plots showed that the amplitudes of the first peak varied slightly (Figures S3–S5). Therefore, the increase in the charge density near the negative electrode in response to ΔΨ was due to the repulsion of anions, not due to the movement of positively charged polymer network towards the positive electrode. Figure 7c,d show that the crowding of the polymer in response to ΔΨ was less pronounced compared with the that of anions on the positive electrode.

### 3.5. Mobility of Ions

The simulations for predicting the diffusion coefficient of the polymerised IL systems were performed at between 300 and 500 K. Using an elevated temperature accelerates the mobility of ions in the electrolyte, and this is a common practice in supercapacitor simulations [58,59]. There are also published work where the mobility of ions in ILs was predicted at room temperature [57,60,61]. The mean-square displacement (MSD) of the anions was calculated at varying temperatures via the following equation:

(1) $$MSD=\frac{1}{N}\sum_{i=1}^{N}\langle {\left(\vec{r_{i}}\left(t\right)-\vec{r_{i}}\left(0\right)\right)}^{2}\rangle$$

where $\vec{r_{i}}\left(t\right)$ and $\vec{r_{i}}\left(0\right)$ are the position of atom *i* at time *t* and time 0, respectively. The MSD was calculated for the anions of IL (*N*) making up the system.

**Figure 8:**
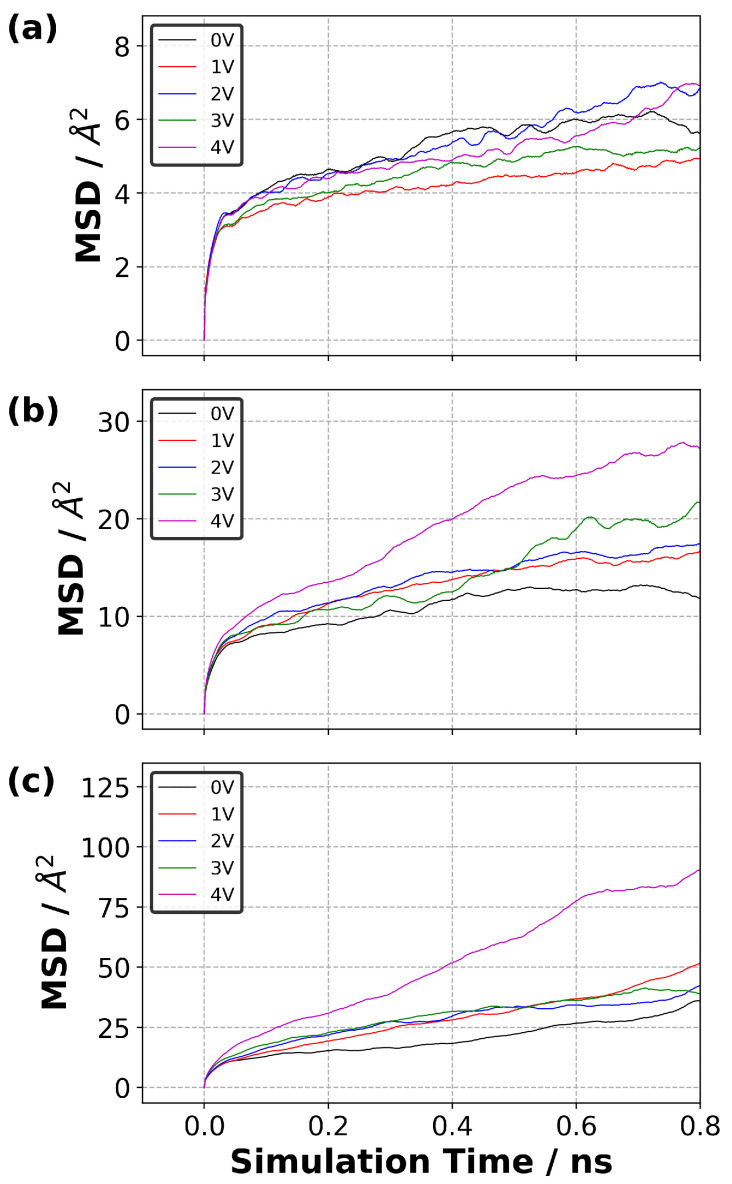
Mean square displacement (MSD) curves for the anion that is found in the electric double layer (EDL) (the thickness of 10 Å from the positive electrode) obtained at different potential difference and temperature: (**a**) 300 K, (**b**) 400 K and (**c**) 500 K.

The results obtained for the anions that were found within a distance of 10 Å from each electrode indicated that the anions were more mobile in the vicinity of the positive electrode (Figure 8) compared with that in the negative electrode (Figure S6). This result is expected because there are more anions accumulated near the positive electrode with an increasing trend in response to the potential difference.

The effect of the value of ΔΨ was less pronounced at the lowest temperature (300 K) tested. A maximum displacement of 5–7 Å${}^{2}$ at a time of 0.8 ns (Figure 8a) was observed. When the temperature increased, the mobility of the anions near the positive electrode increased, and the effect of ΔΨ was more visible. The anions gained increased mobility, ∼28 and 95 Å${}^{2}$ at a time of 0.8 ns, (Figure 8b,c) when the potential difference (ΔΨ) was 4 V at 400 and 500 K, respectively. This can be attributed to the formation of anion-rich region near the positive electrode in which the concentration increased with both temperature and ΔΨ.

## 4. Conclusions

Solid polymer electrolytes (SPEs) are an important type of electrolytes for various applications. In this work, a complete approach, from ion-monomers to solid polymer electrolyte, was devised and used for providing molecular-level explanations for macroscopic properties. The following important observations were made:

- The computational procedure that was devised and used here, generated reliable results. For example, the distribution of each component (i.e., anion, cation (monomer) and hardener) in the liquid precursor mixture was almost symmetrical on both electrodes, indicating a well-equilibrated liquid system in the presence of solid electrodes.
- The normalised mass density distribution (MDD) plots revealed a first peak at a distance of ∼3.5 Å from each electrode for the components in the liquid mixture. This is the shortest distance with the electrodes that the atoms of components can approach due to the strong solid–liquid interactions. In addition, a second peak for the anion was formed at ∼5.5 Å. This is attributed to the absolute charge difference between the anion and cation (the ratio in molecules was 1:2). Another observation related to MDDs was the formation of a depleted region for the hardener near each electrode. This can be attributed to the lack of free space near the electrodes, where both the anion and cation occupied most of the space. Therefore, a rational design of a supercapacitor based on SPE needs to carefully consider the anion/cation/hardener architecture, functionality, net charge on each species and attraction/repulsion between species.
- The charge density distribution (CDD) in the liquid precursor mixture showed a positive peak next to both electrodes, which is due to the presence of the cation-rich region next to the electrodes. Additionally, the first negative peak is considered to be the cumulative effect of the presence of both anion and cation in the vicinity of electrodes. In short, the CDD can be used as one of the informative metrics to better understand and predict the supercapacitor’s performance.
- The distribution of the reacted reactive atomic sites that are found on each component indicated a solidification of the polymer during the polymerisation process and proved the limited mobility of the polymer network. In addition, the solidification of the polymer network did not affect the charge density distribution within the polymer sample with no potential applied. This means that a well-equilibrated liquid precursor mixture determines the distribution of atomic sites in the solid polymer.
- Both ΔΨ and temperature increased the absolute value of the charge density on each electrode. This is attributed to the fact of the reorientation/accumulation of ions in the vicinity of EDL. Additionally, the SPE system came to saturation faster at higher temperatures, which proved the positive effect of temperature on the orientation of the components.
- Anions, which are free to move in the polymer network, because they are not chemically connected to the epoxy network formed by the polymerised monomers and hardeners, showed higher mean square displacement (MSD) compared with that for the epoxy network. This is an expected outcome because the anions, which are smaller when compared to the entire epoxy network, respond to the applied potential difference with higher mobility.

## Supplementary Materials

The following are available at https://www.mdpi.com/article/10.3390/polym14235106/s1, Figure S1: Normalised mass density distribution of the reacted atoms; Figure S2: Charge density distribution; Figure S3: Normalised mass density distribution at 300 K; Figure S4: Normalised mass density distribution at 400 K; Figure S5: Normalised mass density distribution at 500 K; Figure S6: Mean square displacement (MSD) curves at different temperatures;

## Abbreviations

The following abbreviations are used in this manuscript:

SPE	Solid polymer electrolyte
LE	Liquid electrolyte
IL	Ionic liquid
ILM	Ionic liquid monomer
DOC	Degree of crosslinking
MD	Molecular dynamics
CPM	Constant potential method
RDF	Radial distribution function
EDL	Electric double layer
MDD	Mass density distribution
CDD	Charge density distribution
