# Supplementary material for: Rational Design of Solid Polymer Electrolyte Based on Ionic Liquid Monomer for Supercapacitor Applications via Molecular Dynamics Study
Source: Polymers (Basel). 2022 Nov 24;14(23):5106. doi: 10.3390/polym14235106 (PMC9737087; doi:10.3390/polym14235106)
Supplement: Supplementary file 1 [file polymers-14-05106-s001.zip › polymers-1962656-supplementary.pdf]

# Rational Design of Solid Polymer Electrolyte based on Ionic Liquid Monomer for Supercapacitor Applications via Molecular Dynamics Study

Baris Demir,[*,†] Kit-Ying Chan,[‡] and Sébastien Livi[¶]

†*Centre for Theoretical and Computational Molecular Science, The Australian Institute for Bioengineering and Nanotechnology, The University of Queensland, Brisbane, QLD, 4072, Australia*

‡*Department of Aeronautical and Aviation Engineering, The Hong Kong Polytechnic University, Kowloon, Hong Kong SAR.*

¶*Université de Lyon, CNRS, UMR 5223, Ingénierie des Matériaux Polyméres, INSA Lyon, F-69621 Villeurbanne, France.*

E-mail: b.demir@uq.edu.au

# Supplementary Information

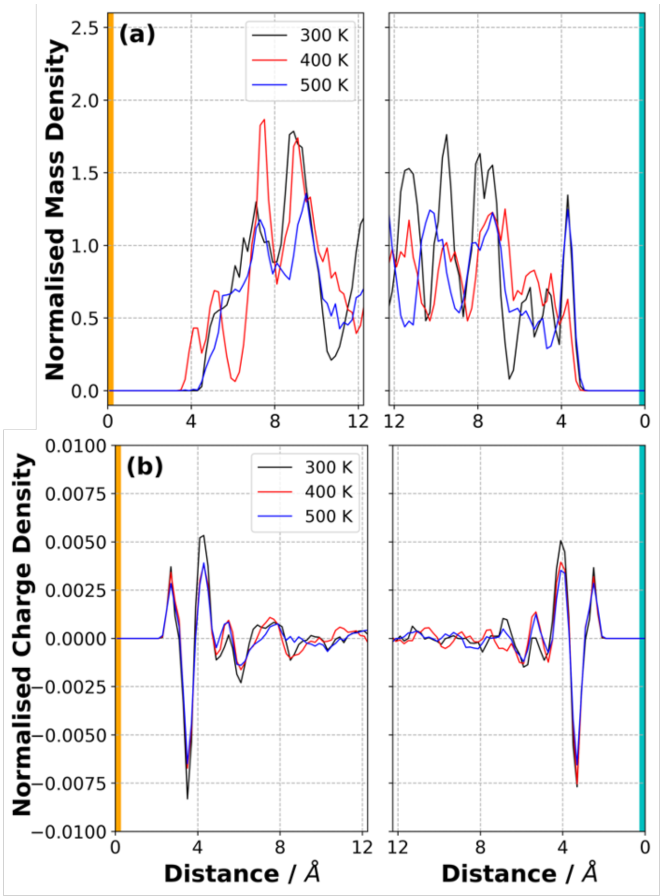


Figure S1: (a) Normalised mass density distribution of the reacted C atoms of cations at various temperatures. (b) Normalised charge density distribution near the negative (red bar) and positive (blue bar) electrodes obtained at the completion of the equilibration process of the polymerised samples at varying temperatures. No potential difference across the simulation cell was applied.

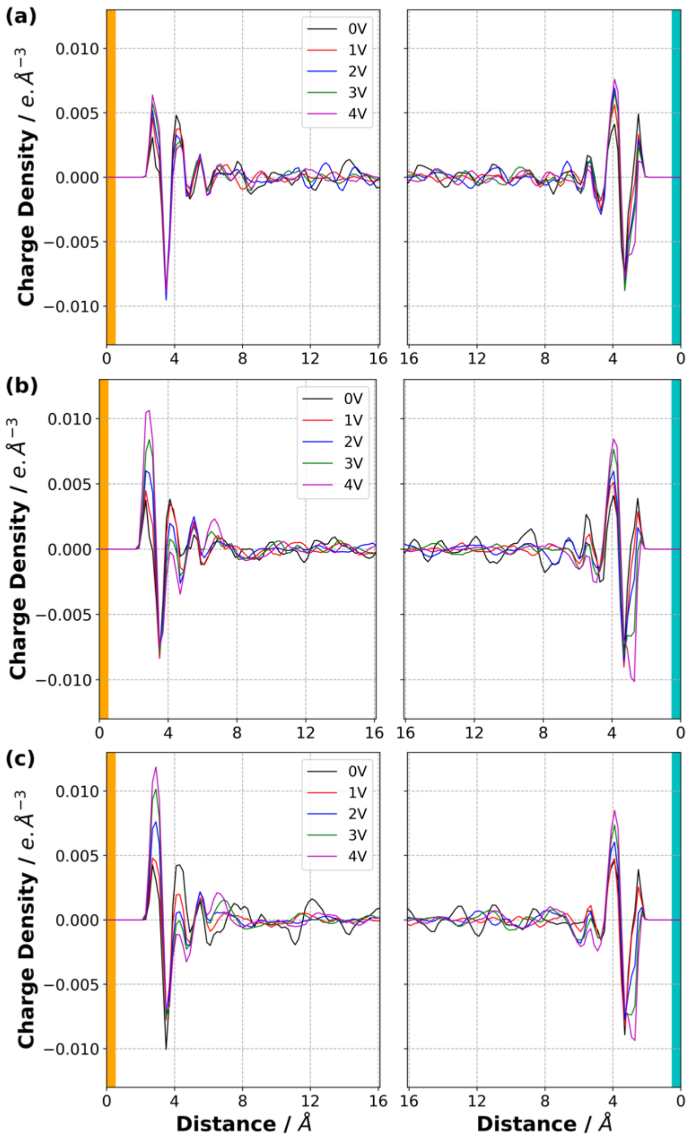


Figure S2: (a) Charge density distribution in the solid polymer electrolyte (SPE) at various $\Delta\Psi$ at (a) 300 K, (b) 400 K and (c) 500 K.

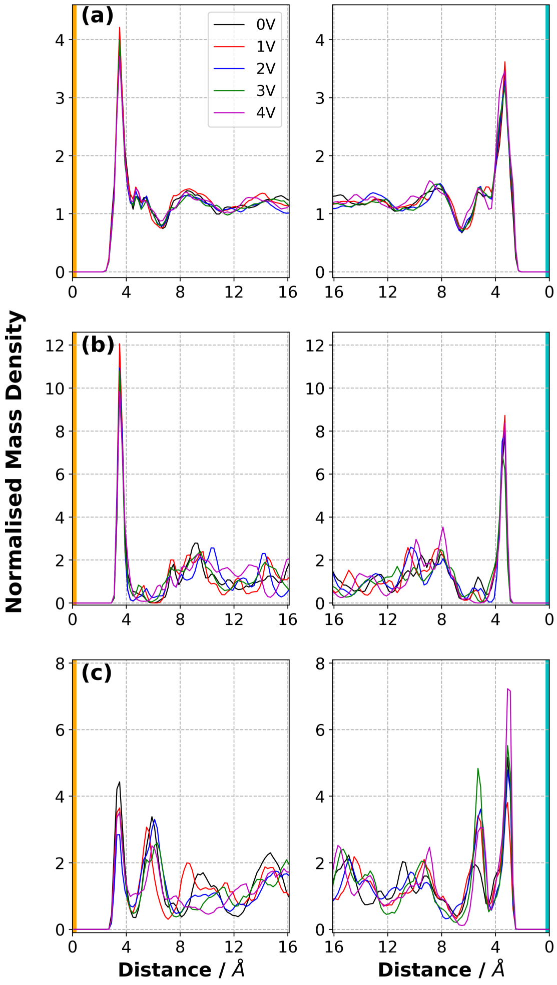


Figure S3: (a) Normalised mass density distribution of the (a) the entire SPE, (b) cations of the SPE, (c) anions at 300 K at various $\Delta\Psi$.

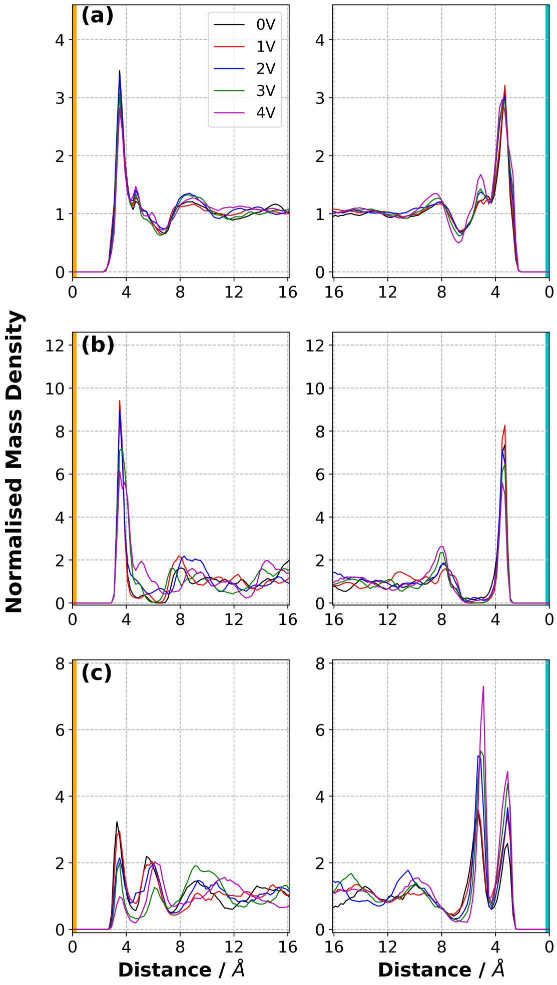


Figure S4: (a) Normalised mass density distribution of the (a) the entire SPE, (b) cations of the SPE, (c) anions at 400 K at various $\Delta\Psi$.

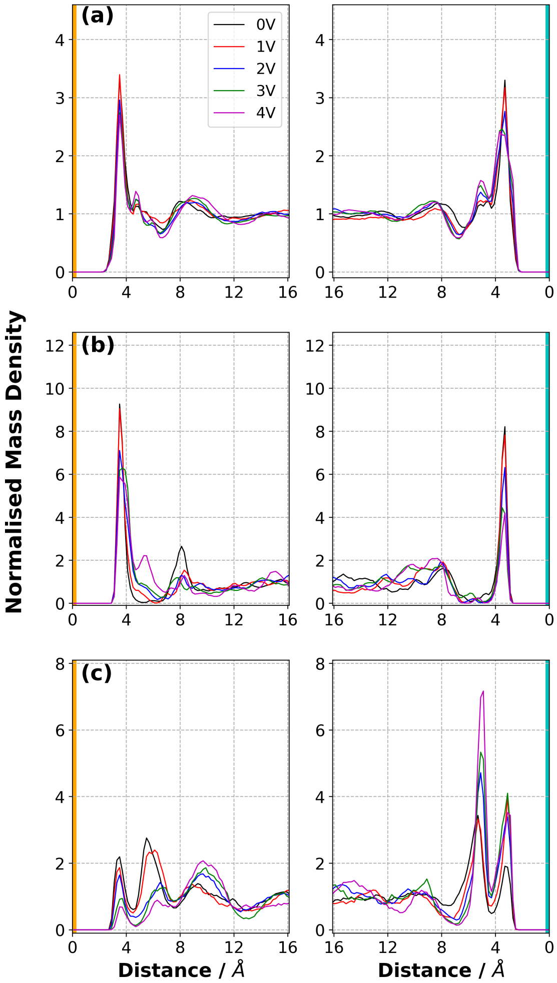


Figure S5: (a) Normalised mass density distribution of the (a) the entire SPE, (b) cations of the SPE, (c) anions at 500 K at various $\Delta\Psi$.

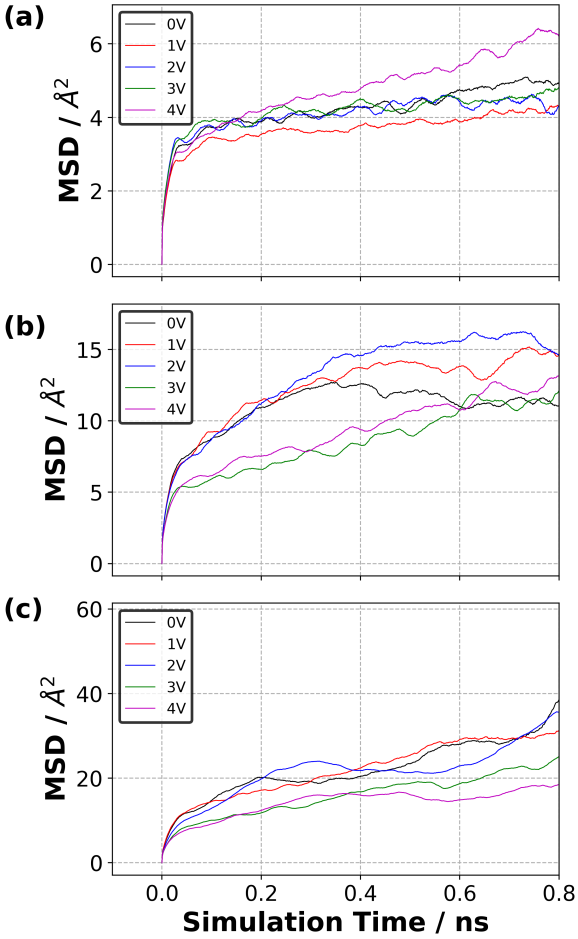


Figure S6: Mean square displacement (MSD) curves for the anion found in the electric double layer (EDL) (the thickness of 10 Å from the negative electrode) obtained at different potential difference and temperatures: (a) 300 K, (b) 400 K and (c) 500 K.
